# PANDORA-seq reveals human sperm sncRNA signature endowed with sperm quality assessment

**DOI:** 10.1016/j.gendis.2025.101807

**Published:** 2025-08-18

**Authors:** Ruofan Huang, Yiting Yang, Wenlin Jiang, Zheng Cao, Junchao Shi, Xiao-Ou Zhang, Yunfang Zhang

**Affiliations:** a Clinical and Translational Research Center of Shanghai First Maternity and Infant Hospital, Shanghai Key Laboratory of Signaling and Disease Research, Frontier Science Center for Stem Cell Research, School of Life Sciences and Technology, Tongji University, Shanghai 200092, China; b NHC Key Lab of Reproduction Regulation, Shanghai-MOST Key Laboratory of Health and Disease Genomics, Shanghai Institute for Biomedical and Pharmaceutical Technologies (SIBPT), Shanghai 200032, China; c China National Center for Bioinformation and Beijing Institute of Genomics, Chinese Academy of Sciences, Beijing 100101, China

**Keywords:** Male subfertility, PANDORA-seq, rsRNA, Sperm quality, tsRNA

## Abstract

One of the leading causes of human subfertility is the continuous decline in semen quality, contributing to a global fertility crisis. Over half of subfertile men suffer from asthenozoospermia and teratozoospermia, with mechanisms still largely unknown. Traditional small noncoding RNA sequencing (sncRNA-seq) primarily targets miRNAs, failing to capture the broader spectrum of small noncoding RNAs (sncRNAs), including abundant transfer RNA-derived small RNAs (tsRNAs) and ribosomal RNA-derived small RNAs (rsRNAs). These sncRNAs possess complex RNA modifications and non-canonical terminal structures, impeding their accurate profiling. In this prospective cohort study, we addressed these limitations by combining our state-of-the-art PANDORA-seq with traditional sncRNA-seq, which generated the most comprehensive sncRNA landscape of human sperm from 25 participants with asthenozoospermia, teratozoospermia, or normozoospermia. PANDORA-seq significantly improved the annotation efficiency of sncRNAs and delivered a more detailed characterization for tsRNAs and rsRNAs, which were strongly correlated with key clinical indicators of sperm quality, thereby enhancing our understanding of the landscape of human sperm sncRNAome and its association with male subfertility. Importantly, machine learning with Lasso regression identified specific tsRNA/rsRNA signatures as highly effective clinical biomarkers (AUC ≥ 0.83) for predicting sperm abnormalities, offering significant improvements over World Health Organization-based semen quality assessments and novel insights for clinical diagnosis.

## Introduction

The decline in fertility rates has become a pressing global issue, particularly prevalent in developed nations and increasingly evident in some developing countries.^1^ This trend is driven by a complex interplay of various factors, including changing social values, environmental conditions, and psychological factors such as lifestyle.^2^ Nevertheless, among couples actively seeking parenthood, infertility rates remain notably high, affecting approximately 15%–20% of this population. Notably, male-related factors account for half of these cases.^3^^,^^4^ Asthenozoospermia (AZS) and teratozoospermia (TZS) are two major clinical manifestations of male subfertility, characterized by reduced sperm motility and abnormal morphology, respectively.^5^ However, current clinical semen analysis provides minimal information about molecular etiologies.^6^ Therefore, a systematic investigation of molecular biomarkers on male fertility would enable more precise assessment of sperm quality and provide mechanistic insights into the molecular basis underlying aberrant sperm parameters.

Small non-coding RNAs (sncRNAs) are highly abundant in mature sperm and play crucial roles in regulating spermatogenesis and sperm function.^7^, ^8^, ^9^, ^10^, ^11^, ^12^, ^13^, ^14^, ^15^, ^16^, ^17^, ^18^, ^19^, ^20^ For instance, the deletion of Piwi-interacting RNA (piRNA)-associated proteins causes male infertility through deleterious effects on piRNA biogenesis.^21^ Moreover, tRNA-derived small RNAs (tsRNAs) in sperm from high-fat-diet-fed mice are involved in the functional regulation of transmitting paternally acquired metabolic disorders to offspring.^14^^,^^17^ Unlike the well-known microRNAs (miRNAs), the biogenesis of tsRNAs and rRNA-derived small RNAs (rsRNAs) involves the cleavage of their tRNA and rRNA precursors by specific ribonucleases.^22^, ^23^, ^24^, ^25^ Accordingly, tsRNAs can be categorized as 5′-tsRNAs, inner′-tsRNAs, and 3′-tsRNAs based on their cleavage sites by Dicer, Angiogenin, and other RNases.^22^, ^23^, ^24^ Likewise, rsRNAs are classified by their derivation from different rRNA origins, such as 5S, 5.8S, 18S, and 28S rsRNAs.^25^^,^^26^ As reported previously, tsRNAs exhibit multifaceted regulatory capacity in various biological processes, which could regulate gene expression at multiple levels, including transcription,^27^^,^^28^ post-transcription,^29^ and translation.^30^, ^31^, ^32^ Additionally, some tsRNAs could modulate retrotransposon activity through diverse mechanisms.^33^ On the contrary, the functions and regulatory mechanisms of rsRNAs are largely unknown, with only limited evidence suggesting their involvement in immune responses.^34^^,^^35^ Consequently, the functional significance of various sncRNAs, particularly rsRNAs, in mature sperm remains largely enigmatic.

The primary challenge in investigating sncRNAs in sperm lies in constructing sncRNA libraries, which requires efficient adaptor ligation and robust reverse transcription of RNAs into complementary DNA (cDNA), a process normally impeded by RNA modifications.^26^ For instance, 3′ end modifications (including 3′-P and 2′,3′-cP) can obstruct adaptor ligation, while RNA methylation modifications, including m^1^A, m^3^C, m^1^G, and m^2^_2_G, hinder efficient reverse transcription.^36^^,^^37^ Consequently, the subsequent sequencing often fails to accurately reflect the actual abundance and distribution of sncRNAs, particularly for those with diverse and abundant modifications, such as rsRNAs and tsRNAs.^26^^,^^38^, ^39^, ^40^ In this study, we employed our previously established sncRNA-seq method, PANDORA-seq (panoramic RNA display by overcoming RNA modification aborted sequencing), to comprehensively profile sncRNAs in human sperm across AZS, TZS, and healthy control samples. We applied a two-step enzymatic treatment of small RNAs with T4 polynucleotide kinase (T4PNK) and α-ketoglutarate-dependent dioxygenase (AlkB) to efficiently remove most modifications that interfere with adaptor ligation and reverse transcription, thereby significantly facilitating reverse transcription during cDNA library construction process. By comparing the results from PANDORA-seq with traditional sncRNA-seq, we analyzed the distribution and abundance of sncRNAs in human sperm samples. Subsequent analyses identified multiple linear correlations between tsRNA/rsRNA levels and the clinical sperm quality parameters. Furthermore, machine learning combined with Lasso regression demonstrated that tsRNAs/rsRNAs had superior capability in identifying aberrant sperm compared with established clinical indicators, serving as a valuable adjunct to the World Health Organization-based semen quality assessment parameters. Overall, our study presents the most comprehensive sncRNA profile in human sperm to date and highlights the potential application of tsRNAs and rsRNAs in clinical sperm quality assessments, providing novel insights to understand the underlying molecular mechanisms of human sperm quality control.

## Materials and methods

### Ethics approval and informed consent

The implementation of all experiments adhered to and was conducted under the relevant provisions outlined in the World Medical Association’s Declaration of Helsinki. The research described in this paper received unanimous approval from the Medical Ethics Committee of Shanghai Institute of Planned Parenthood Research (SIPPR; Shanghai, China; Approval No. PJ2019-24), and each participant provided signed informed consent.

### Participant recruitment and collection/analysis of human semen samples

A total of 25 males (aged 24–34) undergoing semen analysis at the Reproductive Medicine Centre of Zhongshan Hospital (Shanghai, China) were enrolled in this study. All participants were instructed to abstain from ejaculation for 2–7 days before sample collection. Semen samples were collected by masturbation, allowed to liquefy at 37 °C for 30 min, and subsequently subjected to semen analysis. At least 200 spermatozoa were assessed per semen sample to generate the final semen analysis report. All semen samples included in this study were tested negative for white blood cells. According to the guidelines outlined by the World Health Organization (2010, fifth edition) regarding sperm motility and morphology parameters, the semen samples included in this study were categorized into normozoospermia (NZS, as healthy controls; *n* = 9), asthenozoospermia (AZS; *n* = 9), and teratozoospermia (TZS; *n* = 7) groups. The classification criteria were as follows: NZS had a normal sperm morphology of more than 4% and a progressive motile sperm rate of more than 32%; AZS had a normal sperm morphology of more than 4% and a progressive motile sperm rate of less than 32%; and TZS had a normal sperm morphology less than 4%.

### Sperm purification from semen samples

In this study, we implemented somatic cell lysis to minimize somatic RNA contamination of human sperm RNA samples. Initially, 2 mL liquefied semen was mixed with phosphate-buffered saline (PBS) at a volume ratio of 1:1 and centrifuged at 200 *g* for 5 min, followed by the removal of the supernatant. Subsequently, the resuspension of the precipitate was performed with 1 mL PBS, and the centrifugation and resuspension process were repeated. Then, the suspension was filtered through a 70 μm cell strainer (Corning, Harrodsburg, Kentucky, USA) on ice. The mixture was combined with 4 °C pre-chilled cell lysis solution (composed of 0.1% sodium dodecylsulfate, 0.5% Triton X-100, dissolved in sterile 1‰ diethylpyrocarbonate-treated water) at a volume ratio of 1:5 (sperm suspension: cell lysis solution). After gentle inversion of the centrifuge tube, ensuring thorough mixing, the mixture was left to stand on ice for 30 min. Following this, the mixture was again subjected to centrifugation at 600 *g* for 5 min, and the supernatant was discarded. A repeat of the centrifugation and supernatant removal steps was carried out using 1 mL PBS, and the final precipitate was resolved in 1 mL PBS. Subsequently, centrifugation at 10000 g at 4 °C for 5 min yielded a visible pure white precipitate at the bottom of the tube. To maximize the removal of somatic RNA components derived from cell lysis in the supernatant, the supernatant was carefully aspirated and discarded. Finally, 1 mL Trizol reagent (Invitrogen, Waltham, Massachusetts, USA) was added to each white precipitate in a 1.5 mL centrifuge tube for RNA extraction.

### Sperm RNA extraction

A 1 mL syringe (0.45 mm × 15 mm, regular wall and short bevel, Changzhou Yuekang Medical Instruments, China) for repetitive aspiration to disperse and thoroughly mix the solution was used to ensure that the precipitate could smoothly pass through the syringe needle. Subsequently, the mixture was put on ice for 2–3 h, followed by the addition of 0.2 mL chloroform and vigorous shaking, with a subsequent incubation at room temperature for 2–3 min. The mixture underwent centrifugation at 13,000 g and 4 °C for 15 min. The upper aqueous phase was carefully collected into a new 1.5 mL centrifuge tube, to which 600 μL isopropanol and 1–2 μL glycogen (Invitrogen Thermo Fisher AM9510) were added. The centrifuge tube was gently inverted, and the mixture was left to slowly precipitate at −20 °C overnight. Subsequent centrifugation was performed at 13,000 g and 4 °C for 20 min. The precipitate was washed with 75% ethanol and centrifuged at 13,000 g and 4 °C for 10 min. Once the precipitation was air-dried, it was resuspended in 7.5–10 μL of RNase-free water as total RNA, designated for subsequent reverse transcription, PANDORA-seq, and northern blotting experiments.

### Sperm RNA reverse transcription and purity detection

RNA reverse transcription was carried out following Takara’s instructions (catalog number: PR047-A; Iwate, Japan). Briefly, the RNA samples underwent gDNA Eraser treatment at 42 °C for 2 min, reaction mix preparation on ice, and subsequent reverse transcription at 37 °C for 15 min. The RNA was then converted into cDNA at 85 °C for 5 s. Reverse transcription PCR was conducted to evaluate the efficiency of somatic cell lysis by measuring the mRNA expression levels of somatic cell-specific genes, including cadherin-1 (CDH1), cadherin-2 (CDH2), CD4, and the germ cell-specific gene KIT. Also, the sperm-specific gene protamine-2 (PRM2) primers were used for sperm purity assessment. PCR products were analyzed on a 1.5% agarose gel and imaged using a Tanon gel imaging system (mini space 1000, Tanon, Shanghai, China). The primer sequences and PCR procedure are detailed in Table S1.

### Isolation of sncRNAs and PANDORA-seq

PAGE gel was used to isolate sncRNAs as described.^26^ Briefly, total RNA was denatured at 95 °C for 5 min with RNA loading dye (New England Biolabs, B0363S), separated by 15% denatured urea-PAGE, and stained with SYBR Gold (Thermo Fisher, S11494). 15–50 nucleotides (nt) were excised according to RNA ladders (New England Biolabs, N0364S; Takara, 3416), and RNA was extracted. For standard RNA-seq, small RNA library construction and sequencing were directly performed using the Vazyme NR801 kit. For the PANDORA-seq method, the following steps are involved: RNA was incubated with T4PNK at 37 °C for 20 min, and then the product was treated with AlkB at 37 °C for 30 min. These enzyme reaction solutions are displayed in Table S2. TRIzol LS was added to the reaction mixture at a volume ratio of 3:1 (TRIzol LS: reaction mixture). Subsequently, RNA was extracted as described above (see "sperm extraction"). The construction of small RNA libraries, specifically targeting the 15–50 nt RNA fraction, was carried out using the VAHTS Small RNA Library Prep Kit for Illumina (Vazyme, NR801), followed by sequencing performed by Sequanta Technologies (Shanghai, China).

### Small RNA sequencing data processing

Raw sequencing reads were processed and annotated using the SPORTS software (version 1.1) with up to one mismatch allowed (parameter: -M 1).^40^ After preprocessing, reads ranging from 18 to 45 nt in length were retained for further analysis. Briefly, reads were sequentially aligned to the human reference genome (GRCh38/hg38) and the following non-coding RNA datasets, the rRNA and Y RNA from NCBI, the genomic tRNA from GtRNAdb, the mitochondrial tRNA from mitotRNAdb, the miRNA from miRBase, the non-coding RNA from Ensembl ncRNA database and Rfam, and the piRNA from piRBase, to estimate individual sncRNA expression. sncRNA transcripts with at least one read in at least 50% of the samples were retained as the expressed sncRNAs for further analysis. Unless otherwise specified, sncRNA transcript expression was normalized to values for reads per million mapped reads. For tsRNAs and rsRNAs, sncRNA transcripts were further categorized into individual subgroups based on their respective parent RNAs of origin.

### Identification of differentially expressed sncRNAs

Differentially expressed sncRNAs were identified using the DESeq2 package (v1.34.0). We compared sncRNA expression profiles between two groups: AZS versus NZS, and TZS versus NZS. Significance for differentially expressed sncRNAs was defined by an adjusted *P*-value threshold of ≤ 0.05 and an absolute log_2_-fold change of ≥ 1.5.

### Northern blotting and PCR validation

For the northern blotting, total RNA was denatured at 95 °C for 5 min. One μL of each sample was loaded and separated by 15% urea-PAGE and stained with SYBR Gold. After ultraviolet imaging, we adjusted the load volume of each sample for normalization and repeated gel electrophoresis, SYBR Gold staining, and ultraviolet imaging. Then, the gel was transferred to a Nytran SuperCharge membrane (Schleicher and Schuell, 32-10416296) and crosslinked with 0.12 J of ultraviolet. Subsequently, the membrane was pre-hybridized in DIG pre-hybridization solution (Roche: DIG Easy Hyb, 11603558001) at 42 °C for 30 min. The membrane was then incubated at 42 °C overnight (12–16 h) with synthesized DIG-labeled oligonucleotide probes at a final concentration of 16 nM. The probe sequences are provided in Table S3. Following overnight hybridization, the membrane was washed twice at 42 °C with low-stringent buffer (2 × SSC with 0.1% (w/v) sodium dodecylsulphate) for 15 min each, followed by two washes with high-stringent buffer (0.1 × SSC with 0.1% (w/v) sodium dodecylsulphate) for 5 min, and a final rinse in washing buffer (1 × SSC) at room temperature for 10 min. The membrane was transferred to 1 × blocking buffer (Roche, 11096176001) and incubated at room temperature for 3 h. Subsequently, the DIG antibody (Roche: Anti-Digoxigenin-AP Fab fragments, 11093274910) was added to the blocking buffer at a ratio of 1:10,000 and incubated at room temperature for an additional 30 min. The membrane was then washed four times in DIG washing buffer (1 × maleic acid buffer, 0.3% Tween-20) for 15 min each, rinsed in DIG detection buffer (0.1 M Tris–HCl, 0.1 M NaCl, pH 9.5) for 5 min, and incubated with CSPD ready-to-use reagent (Roche, 11755633001) in the dark at 37 °C for 15 min before imaging using a Tanon imaging system (Shanghai, China).

For reverse transcription PCR, total RNA was treated with T4PNK and AlkB sequentially as described before. Then, following Takara’s instructions (Iwate, Japan, catalog number: PR047-A), reverse transcription was performed to obtain cDNA. The validation of sncRNA was conducted using PCR. The procedure is detailed in Table S1, and the primers used are displayed in Table S3.

### Correlation analysis between sncRNA types with clinical semen parameters

Spearman correlation coefficients were calculated to assess the relationships between various sncRNA types and two key aspects of sperm quality: motility and morphology. Motility parameters included the percentage of immotile (IM), progressively motile (PR), and non-progressively motile (NP) sperm. Morphology parameters assessed the percentage of sperm with abnormal head/tail morphology (Abnormal), intact heads (Total_head), intact necks (Total_neck), cytoplasmic droplets (CD), head shape index (TZI), and a strict morphology index (SDI). Additionally, for selected sncRNA subcategories, we employed linear regression using the lm function in the R package to further investigate the association between their expression levels and specific clinical indicators.

### Development of the sncRNA signature score using machine learning

To identify a robust and informative sncRNA signature for male subfertility diagnosis, we employed a machine learning approach. First, we selected candidate features by focusing on differentially expressed sncRNAs (*p* < 0.05) with a minimum average expression level of 10 reads per million and a strong correlation coefficient (*r* > 0.5) with semen parameters. Next, we utilized the LASSO regression model implemented in the R package with the cv.glmnet function for feature selection and model building. The LASSO method shrinks regression coefficients, effectively removing features with minimal contribution to the model and preventing overfitting. Cross-validation was employed to optimize the model and identify the optimal lambda parameter. Features with non-zero coefficients in the final model were considered the core sncRNAs for the signature. The sncRNA signature scores were constructed by weighting the expression values of selected sncRNAs with their LASSO regression coefficient values. Finally, to evaluate the performance and generalizability of our model, we employed the Random Forest algorithm to compare the feature importances and receiver operating characteristic (ROC) curves of the sncRNA signature score, individual sncRNAs, and clinical indicators in distinguishing different disease states.

### Statistical analysis

Statistical significance was assessed using Student’s *t*-test for normally distributed data and the Wilcoxon rank sum test for non-parametric data. Heatmaps for tsRNA subcategory analyses were generated using the pheatmap function from R (version 4.1) after log_2_ transformation of the data. Spearman correlation coefficients were used to assess correlations between tsRNA categories.

## Results

### PANDORA-seq enhances the detection of sncRNAs in human sperm

**Figure 1:**
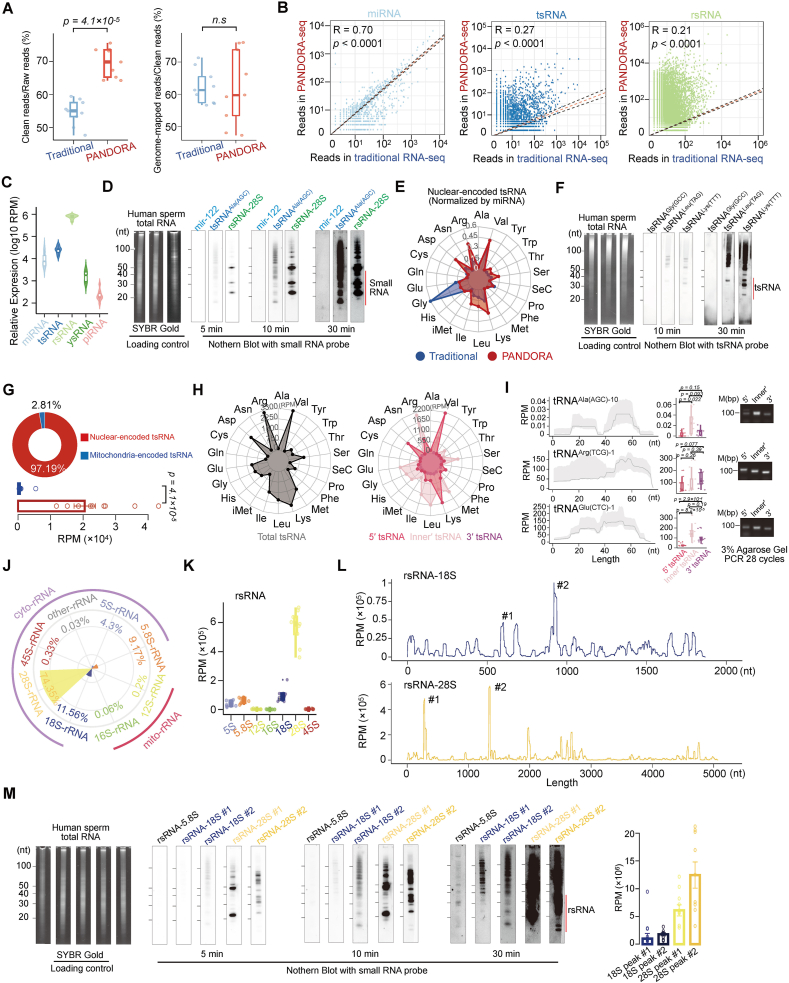
Characterization and comparison of human sperm sncRNA signatures between traditional sncRNA-seq and PANDORA-seq. **(A)** Comparison of the relative ratio of clean reads to raw reads and reads aligned to the genome to clean reads between traditional sncRNA-seq and PANDORA-seq. **(B)** The scatter plots depicting the correlation of miRNA, tsRNA, and rsRNA expression profiles of paired traditional sncRNA-seq and PANDORA-seq. Spearman correlation coefficients and *P*-values are shown. **(C)** Comparison of the relative expression levels of four major sncRNA origins (miRNA, tsRNA, rsRNA, ysRNA, and piRNA) in healthy human sperm determined by the PANDORA-seq protocol. **(D)** Expression levels of miRNAs, tsRNAs, and rsRNAs in human sperm samples validated by northern blotting. **(E)** The radar plot showing the different relative expression proportions of each tsRNA subcategory with respect to the two protocols. **(F)** Northern blotting validation of tsRNA^Leu(TAG)^, tsRNA^Lys(TTT)^, and tsRNA^Gly(GCC)^ in human sperm. **(G)** Visualization of the proportional distribution of cyto-tsRNAs and mt-tsRNAs in healthy human sperm determined by the PANDORA-seq protocol. **(H)** The radar plots illustrating the relative expression proportions of each tsRNA subcategory in relation to three distinct tsRNA origins (5′, inner′, and 3′). **(I)** Sequence mapping location (left), expression profile (middle), and reverse transcription PCR validation (right) of tsRNA^Ala(AGC)^, tsRNA^Arg(TCG)^, and tsRNA^Glu(CTC)^ in human sperm. **(J)** Distribution of expression proportions for six nucleus-encoded rsRNAs and two mitochondria-encoded rsRNAs. **(K)** The boxplots showing the relative expression of five nucleus-encoded rsRNAs and two mitochondria-encoded rsRNA categories. **(L)** Visualization of expression signature and sequence mapping location of rsRNA-18S and rsRNA-28S in human sperm samples. **(M)** Northern blotting validation of rsRNA-5.8S, selected rsRNA-18S main peaks (peak #1, peak #2), and selected rsRNA-28S main peaks (peak #1, peak #2) in human sperm samples.

To unveil the sncRNA profile in human sperm, purified human sperm were used to avoid somatic cell contamination (Fig. S1A). Both PANDORA-seq and traditional sncRNA-seq were performed with human sperm RNA from nine NZS samples. Compared with traditional sncRNA-seq data, PANDORA-seq captured more effective sncRNA reads at both clean and genome-mapped levels (Fig. 1A), and identified over fifteen distinct sncRNA categories (Fig. S1C and S1D) in human sperm. Notably, PANDORA-seq revealed measurable enrichment of tsRNAs and rsRNAs in human sperm, while miRNAs exhibited a similar expression pattern between the two sequencing methods (Fig. 1B; Fig. S1E and S1F). Northern blotting experiments validated the three top-expressed small RNAs within each sncRNA subtype, and confirmed that the abundance of top-expressed rsRNA was above the top-expressed tsRNA and miRNA, indicating the high enrichment of rsRNA in human sperm, consistent with the PANDORA-seq results (Fig. 1C and D). Additionally, piRNAs showed some enrichment in PANDORA-seq (Fig. S1B), although their overall expression levels were relatively low (Fig. 1C).

With PANDORA-seq, both nuclear-encoded and mitochondrial tsRNAs (normalized to miRNAs as previously reported^26^) in human sperm showed distinct expression signatures compared with traditional sncRNA-seq (Fig. 1E and F; Fig. S2A–S2E). Notably, PANDORA-seq showed higher mapping efficiency on inner′-tsRNAs in human sperm (compared with 5′ and 3′ with CCA tail, classified based on the cleavage position of tRNA) (Fig. 1G and H; Fig. S2F, S2G, S2J, S2K), along with a strong tRNA isotype bias of origination position (Fig. 1H and I). Having characterized tsRNA expression profiles, we subsequently investigated the expression landscape of rsRNAs in human sperm. Consistent with the sncRNA profile in mouse sperm revealed by PANDORA-seq,^26^ rsRNA was also the most abundant sncRNA in human sperm (Fig. S2H and S2I). Among these rsRNAs, the majority of rsRNAs originated from cytoplasmic rRNA, with only a minor contribution from mitochondrial rRNA (Fig. 1J). Moreover, rsRNA-28S (74.35%) and rsRNA-18S (11.56%) together constituted over 85% of the total rsRNA population, followed by 5.8S-derived rsRNAs contributing a substantial 9.17% (Fig. 1J and K). Northern blotting validation confirmed that, in the cellular context, rsRNAs derived from 5.8S, 18S, and 28S rRNAs displayed a stepwise increase in expression levels, mirroring the sequencing data (Fig. 1M). Interestingly, rsRNAs presented specific sequence origins and distribution patterns from 28S to 18S rRNAs (Fig. 1L). Two dominant sequence peaks identified in the sequencing data were further confirmed by northern blotting analysis (Fig. 1M).

### PANDORA-seq unveils distinct sncRNA landscape in subfertile sperm

**Figure 2:**
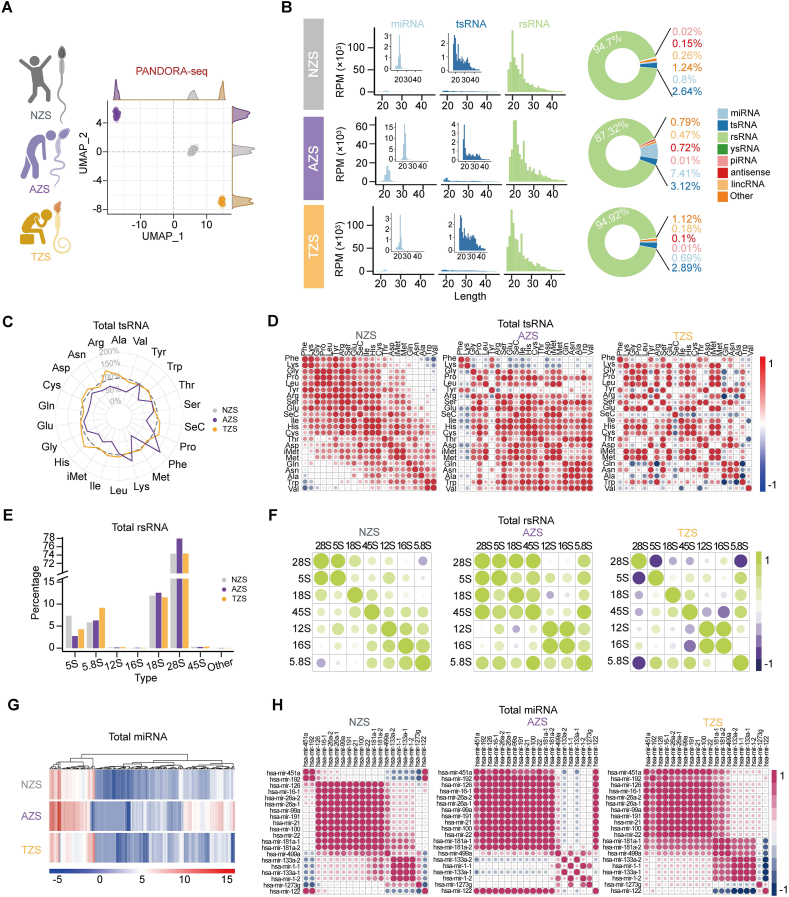
PANDORA-seq unveils sncRNA alteration signatures in AZS and TZS compared with NZS. **(A)** The uniform manifold approximation and projection (UMAP) plot visualizing the distribution of human sperm total sncRNAs among healthy controls (NZS, *n* = 9), AZS samples (*n* = 9), and TZS samples (*n* = 7). **(B)** Dynamic landscapes and length distributions of miRNAs, tsRNAs, and rsRNAs detected. Zoomed panels of miRNA and tsRNA are shown on the plot for clearer visualization. **(C)** The radar plot showing the expression proportions (normalized by NZS samples) of each tsRNA subcategory in AZS and TZS samples. **(D)** The heatmap showing Spearman correlation coefficients between tsRNA subcategory abundances. **(E)** The bar charts showing the expression proportions of six nucleus-encoded rsRNAs and two mitochondria-encoded rsRNAs among NZS, AZS, and TZS. **(F)** The heatmap showing Spearman correlation coefficients between the rsRNA origin abundances. **(G)** The heatmap showing expression profile changes in miRNAs based on a log2-transformed scale among NZS, AZS, and TZS. **(H)** The heatmap showing Spearman correlation coefficients between the top 20 miRNA family abundances.

To further explore the alteration of sncRNA in human abnormal spermatozoa, we recruited patients diagnosed with AZS and TZS, collecting sperm samples for PANDORA-seq to compare them with those from NZS individuals. Unsupervised dimensionality reduction using uniform manifold approximation and projection (UMAP) visualization revealed that PANDORA-seq enabled clear differentiation among the clinical states of the sperm samples (Fig. 2A). In line with our observations in healthy controls, AZS and TZS samples detected over 15 types of sncRNAs (Fig. 2B; Fig. S3A and S3B). The major expression alteration was in rsRNAs, followed by miRNAs and tsRNAs (Fig. 2B; Fig. S3C). For tsRNAs, the AZS samples exhibited a general trend of lower abundance of tsRNA expression across most amino acid-derived tsRNAs, while TZS samples displayed comparable tsRNA levels, compared with NZS samples (Fig. 2C). Notably, the co-expression analysis of tsRNAs among NZS, AZS, and TZS samples showed remarkably different expression patterns (Fig. 2D). Similar expression patterns were also observed in rsRNAs and miRNAs (Fig. 2E–H), revealing dynamic expression alterations at both single sncRNA and overall sncRNA co-expression network levels across different clinical states of human sperm.

### tsRNA expression in subfertile spermatozoa correlates with sperm motility and morphology

**Figure 3:**
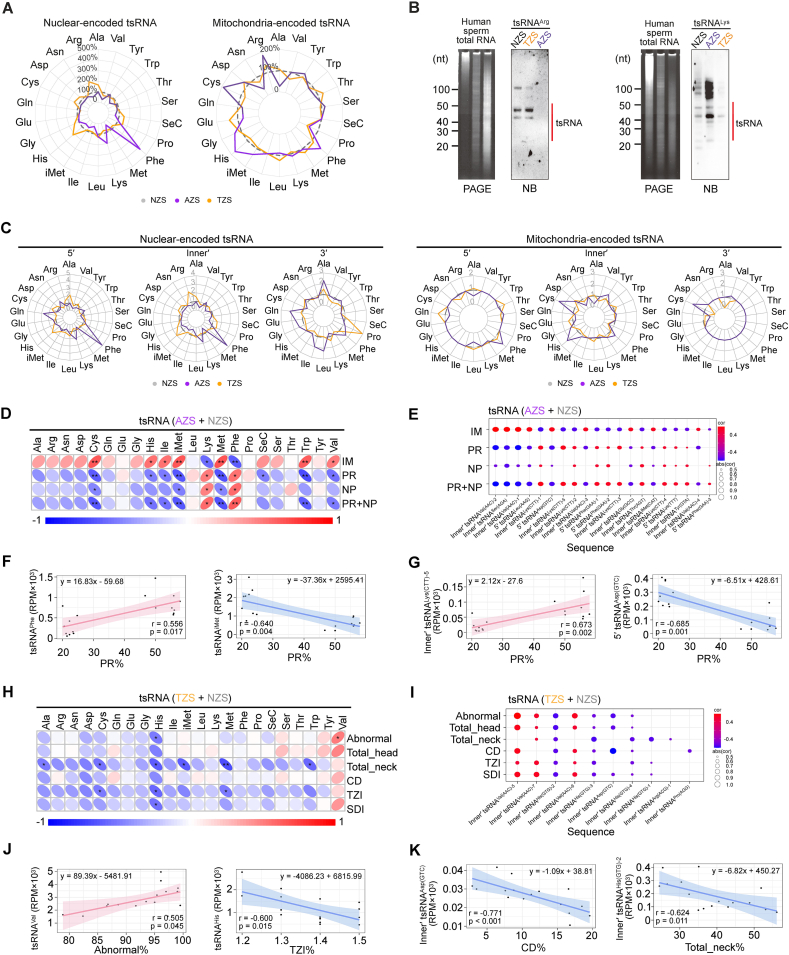
Comprehensive analysis of the expression patterns of tsRNAs in AZS and TZS compared with NZS. **(A)** The radar plots illustrating the relative proportions of nucleus-encoded and mitochondrial tsRNA subcategories in AZS and TZS samples, normalized to NZS. **(B)** Northern blotting validation of tRNA-Arg and tRNA-Lys expression between subfertile (AZS or TZS) and healthy control (NZS) sperm samples. **(C)** The radar plots showing the relative expression levels of nucleus-encoded and mitochondria-encoded tsRNA origins for each tsRNA subcategory in AZS and TZS samples, normalized to NZS. **(D)** Spearman correlation analysis between each tsRNA subcategory abundance and sperm motility. **(E)** The dot plot displaying correlations between individual tsRNA expression levels and sperm motility. **(F)** Representative linear correlations between tsRNA subcategories and sperm motility. **(G)** Representative linear correlations between tsRNA and sperm motility. **(H)** Spearman correlation analysis between each tsRNA subcategory abundance and morphology metrics. **(I)** The dot plot displaying correlations between individual tsRNA expression levels and sperm morphology metrics. **(J)** Representative linear correlations between tsRNA subcategories and sperm morphology metrics. **(K)** Representative linear correlations between tsRNA and sperm morphology metrics. IM, immotility; PR, progressively motility; NP, non-progressively motility; Abnormal, abnormal head/tail morphology; Total_head, intact heads; Total_neck, intact necks; CD, cytoplasmic droplets; TZI, head shape index; SDI, a strict morphology index.

Although there were no evident differences in the overall expression abundance of nuclear or mitochondrial tsRNAs across different disease states (Fig. S4A), we found that the nuclear-encoded tsRNAs from the 5′ end predominated across different disease states, whereas mitochondrial tsRNAs were primarily skewed towards generating inner′-tsRNAs (Fig. S4C). Alterations were detected across different amino acids of tsRNAs among different disease states (Fig. 3C; Fig. S4C and S4D). Notably, a remarkable increase in tsRNA^Lys^ and tsRNA^Phe^ was observed in AZS samples, along with elevated levels of a few tsRNA categories in the TZS samples compared with NZS samples (Fig. 3A). Northern blotting validation confirmed the PANDORA-seq data for nuclear-encoded tsRNA^Arg^, showing higher levels in TZS and lower levels in AZS (Fig. 3B; Fig. S4B). Similarly, the expression trend of nuclear-encoded tsRNA^Lys^, higher in AZS and lower in TZS, was validated by northern blotting analysis, reinforcing the sequencing observations. Mitochondria-encoded tsRNAs also exhibited dynamic variations in subfertile sperm samples (Fig. 3A).

To further explore the clinical indications of sncRNAs in human sperm, we investigated the relationships between various sncRNA types in sperm and clinical semen parameters, including sperm motility (IM, PR, and NP) and sperm morphology (Abnormal, Total_head, Total_neck, CD, TZI, and SDI). Interestingly, miRNA subtypes generally correlated with motility parameters (Fig. S5A and S5B), while sequence–level correlations with morphology were less measurable (Fig. S5C and S5D). Regarding tsRNAs, we found that the overall abundance of specific tsRNA subcategories was closely associated with sperm motility, such as the PR and IM parameters. Specifically, tsRNA^Phe^ and tsRNA^Lys^ positively correlated with PR but negatively correlated with IM, suggesting their potential roles in promoting sperm motility (Fig. 3D–F). On the contrary, other tsRNA subcategories, including tsRNA^iMet^, tsRNA^Met^, tsRNA^Trp^, tsRNA^His^, tsRNA^Ile^, tsRNA^Cys^, and tsRNA^Val,^ displayed negative correlations with PR but positive correlations with IM (Fig. 3D), indicating these tsRNAs might be associated with decreased sperm motility. While correlations between tsRNA subcategories and sperm morphology parameters were less pronounced, some notable associations were observed (Fig. 3H–J). For instance, tsRNA^Val^ showed a positive correlation with the Abnormal indices, while tsRNA^His^ exhibited a negative correlation with Abnormal, Total_neck, TZI, and SDI indices. At the sequence level, individual tRNA sequences displayed a broader range of robust correlations with single or multiple clinical indicators, further supporting the overall trends observed for tRNA subcategories (Fig. 3E–G, I, K).

### rsRNAs show distinct expression patterns in subfertile sperm

**Figure 4:**
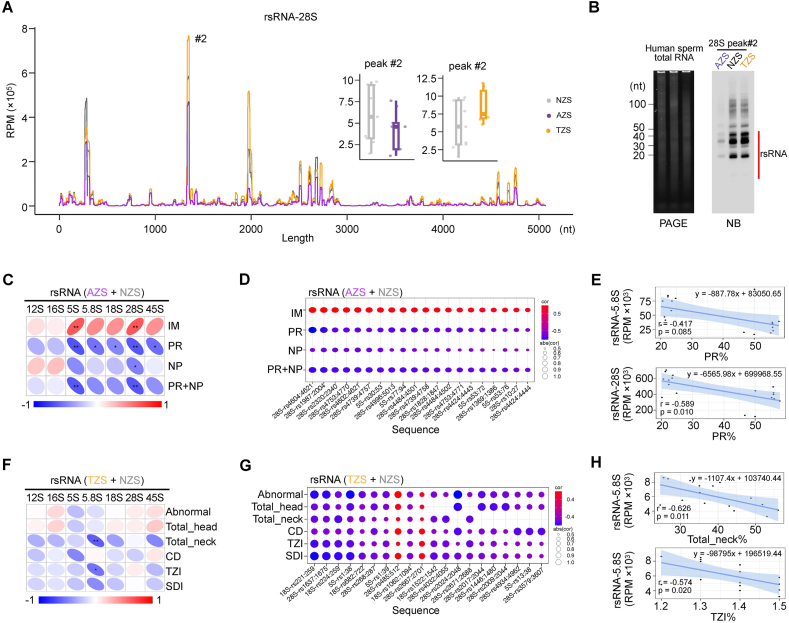
Comprehensive analysis of the expression patterns of rsRNAs in AZS and TZS compared with NZS. **(A)** Sequence mapping location and expression profile of rsRNA-28S between subfertile sperm (AZS or TZS) and healthy control (NZS). **(B)** Northern blotting for the representative expression peaks of rsRNA-28S (peak #2) performed between subfertile sperm (AZS or TZS) and healthy control (NZS) samples. **(C)** Spearman correlation coefficients between the expression abundance of rsRNAs from different origins and sperm motility metrics. **(D)** The dot plot showing sperm motility correlated with individual rsRNA expression levels. **(E)** Representative linear correlations between rsRNAs from different origins and sperm motility metrics. **(F)** Spearman correlation coefficients between the expression abundance of rsRNAs from different origins and sperm morphology metrics. **(G)** The dot plot showing sperm morphology metrics correlated with individual rsRNA expression levels. **(H)** Representative linear correlations between rsRNAs from different origins and sperm morphology metrics.

By mapping and comparing the overall expression profiles of different rsRNAs, we characterized the expression alterations of rsRNAs in human sperm at the resolution of sequence origins from their parental rRNAs (Fig. S6A–S6C; Fig. 4A). All of these rsRNAs that derived from 5S, 5.8S, 18S, and 28S rRNAs exhibited considerable alterations in the AZS and TZS group at different locations from their parental 5S, 5.8S, 18S, and 28S rRNAs. Interestingly, we found a general trend of lower expression abundance of overall 28S rRNA derived rsRNAs compared with healthy controls (Fig. 4A). Specifically, peak #2 of rsRNA-28S exhibited a significant decrease in the AZS group and a rise in the TZS group, which were also validated by northern blotting (Fig. 4B). Moreover, we found that the abundance of rsRNA-28S exhibited strong correlations with all motility parameters (IM, PR, and NP) in the AZS samples compared with other subtypes of rsRNAs (Fig. 4C and D). Interestingly, most 28S-rsRNAs show significant negative correlations with PR, while positively correlated with IM, suggesting a potential role for 28S-rsRNA in regulating sperm motility. In contrast to motility, the correlation profile between rsRNAs from different origins and sperm morphological parameters was generally weak, with only rsRNA-5.8S showing a notable negative correlation with both Total_head and TZI indices (Fig. 4F–H). The increase of rsRNA-5.8S in TZS (Fig. 2D) suggests a potential role in subfertile sperm morphology. Beyond these overall trends, individual rsRNAs displayed distinct responses, with rsRNA-18S, rsRNA-28S, and rsRNA-5S exhibiting significant linear correlation with single or multiple clinical indicators (Fig. 4D–G; Fig. S6D and S6E).

### Sperm sncRNA-based signatures as robust biomarkers in sperm quality evaluation

While sncRNAs have emerged as promising tools for various diseases, their potential in diagnosing human male subfertility syndromes remains largely unexplored. In the context of male subfertility, sncRNAs exhibit distinct patterns across different disease states and demonstrate strong correlations with clinical semen parameters (Figure 2, Figure 3, Figure 4), suggesting their potential as diagnostic biomarkers.

**Figure 5:**
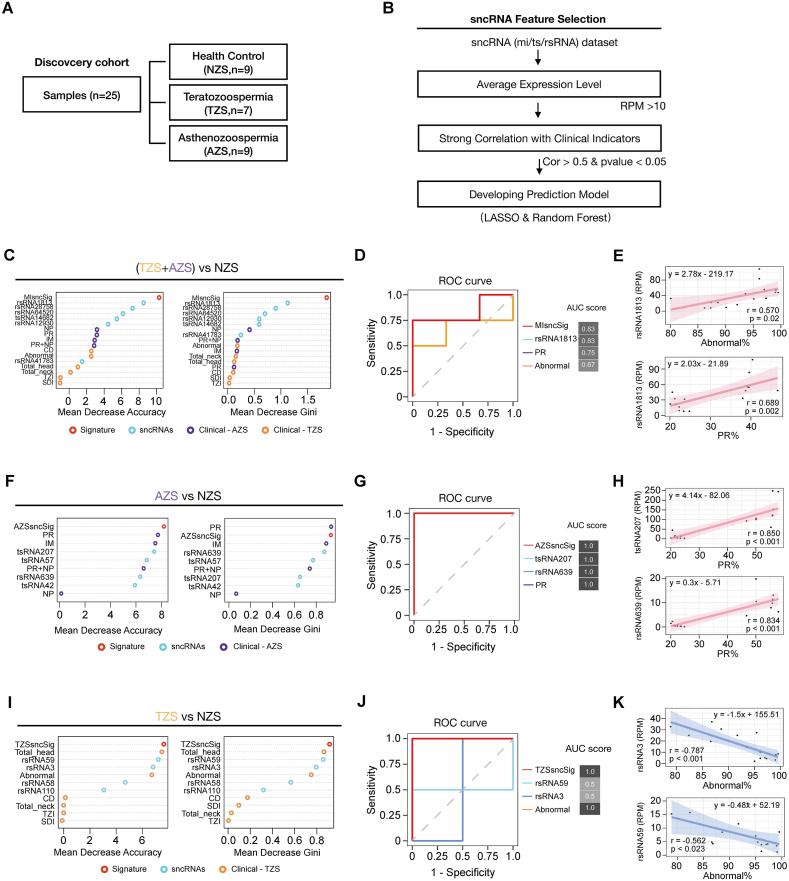
The sncRNA-based signatures robustly distinguish subjects with subfertile sperm from healthy controls. **(A)** The discovery cohort of the study. **(B)** Strategies and workflow of screening sncRNA characteristics for developing the prediction model between subfertile sperm (AZS or TZS) and healthy control (NZS) samples. **(C)** The ranking results of the random forest classifiers for distinguishing subfertile sperm (AZS and TZS) cases from healthy controls, ordered by feature importance. **(D)** The receiver operating characteristic (ROC) curve for sncRNA signatures used to distinguish subfertile sperm (AZS and TZS) cases from healthy controls, along with the corresponding area under the ROC curve (AUC) score. **(E)** Representative linear correlations between selected sncRNA sequences and sperm motility or sperm morphology metrics. **(F, I)** The ranking results of the random forest classifiers for distinguishing AZS (F) or TZS (I) cases from healthy controls, ordered by feature importance. SncRNA signatures, sperm morphology indicators, sperm motility indicators, and individual sncRNAs were marked as red, orange, purple, and blue points, respectively. **(G, J)** ROC curve for selected sncRNA signatures used to distinguish AZS (G) or TZS (J) cases from healthy controls, along with the corresponding AUC scores. **(H, K)** Representative linear correlations between selected sncRNA sequences and sperm motility (H) or sperm morphology metrics (K).

Traditionally, clinical assessment of AZS and TZS relies on evaluating separate clinical indicator metrics derived from semen analysis reports. Here, we aimed to identify one or a group of sncRNAs as potential signatures that could distinguish between normal subjects and male subfertility patients. To further investigate this potential, we employed machine learning approaches to develop and assess classification models. A total of 25 samples were included, comprising healthy controls (NZS samples, *n* = 9), TZS samples (*n* = 7), and AZS samples (*n* = 9). These samples were randomly divided into training (70%) and validation (30%) datasets (Fig. 5A).

At first, we combined samples from patients with AZS and TZS as the male subfertility group by merging expression matrices. We screened sequences that were significantly correlated with sperm motility and morphology parameters to identify candidate sncRNA markers (Fig. 5B). We then used the LASSO method to compress feature vectors and established models for distinguishing male subfertility samples from the control group (Fig. S7A). We finally identified a panel of candidate sncRNAs designated as the male subfertility sncRNA signature (MSsncSig), including five rsRNAs and one tsRNA (Fig. S7B). MSsncSig and the majority of candidate sncRNAs consistently ranked superior to established clinical indicators in differentiating male subfertility samples from the healthy controls (Fig. 5C), with further evidence showing robust diagnostic performance (Fig. 5D and E). These findings suggest that sncRNA signatures hold promise as a novel and potentially more convenient approach for male subfertility diagnosis.

Next, we identified differentially expressed sncRNAs among different conditions and applied a minimum expression threshold to filter out low-expressed ones (Fig. S7C). To systematically evaluate the classification power of the sncRNA signatures, we established an AZS-related sncRNA signature (AZSsncSig) score by weighting the expression values of selected sncRNAs based on their regression coefficient values (Fig. S7D). In the AZS versus NZS group, we ultimately selected four sncRNAs (Fig. S7E). When combined with clinical indicators in the training features assessed with the random forest method, the AZSsncSig outperformed all other indicators (Fig. 5F–H). A similar strategy was employed for the TZS versus normal comparison. Four sncRNAs were selected and formed the TZS-related sncRNA signature (TZSsncSig) score, which ranked higher than the Abnormal and Total_head indicators commonly used for diagnosing TZS (Fig. 5I–K). This demonstrates that the identified sncRNA signatures offered a novel approach for assessing sperm morphology or motility, potentially serving as strong predictors of fertility potential in semen samples.

## Discussion

The discovery of RNA in mammalian sperm during the 1990s challenged the long-held assumption that sperm were transcriptionally inert. Subsequent advances in sequencing technologies have since fueled extensive research into the functional roles of sperm RNA.^41^ However, the comprehensive profiling of sncRNAs in human sperm has been impeded by technical limitations of traditional sncRNA-seq, which are inefficient at capturing sncRNAs with abundant RNA modifications and noncanonical terminal phosphorylation.^26^ In this study, we employed our previously established PANDORA-seq strategy, which enables robust detection of modified sncRNAs, to comprehensively characterize the small RNA profile in human sperm, revealing an underappreciated small RNA landscape with high abundances of rsRNAs and tsRNAs. Our profiling data showed that the actual levels of miRNAs (0.8% in NZS) and piRNAs (0.02% in NZS) were negligible in human sperm. In fact, rsRNAs (94.7% in NZS) and tsRNAs (2.64% in NZS) constituted the predominant small RNA species (> 90%). Although the biological functions of tsRNA and rsRNA in human sperm quality control are still unclear, their abundances in sperm might indicate a new research direction to investigate the underlying regulatory mechanism of male infertility. However, when studies aimed to identify functional miRNAs and other low-abundance small RNAs in sperm, cautions should be taken to avoid potential biases in downstream analyses. Methodological optimization and data sequencing depth improvement might enhance the detection sensitivity of PANDORA-seq for scarce sncRNA types in human sperm and strengthen the data reliability for downstream analyses.

Additionally, we noticed that the overall percentages of clean and mapped reads in the current study were relatively low, which might be attributed to the intrinsic complexity of human semen samples. Nevertheless, we performed stringent quality filtering criteria and rigorous quality control measures to ensure that only high-confidence reads were retained for subsequent analyses during the data preprocessing stage. Although PANDORA-seq enhanced the detection efficiency of these sncRNA types and yielded reproducible results across multiple human sperm samples, further studies with enlarged sample numbers are still required to enhance our understanding of the human sperm sncRNA profile. Moreover, since each sncRNA sequence count is normalized to the sequencing depth (reads per million or RPM), the resulting values actually represent the relative abundance of each sncRNA sequence among human sperm total sncRNAs. The normalization leads to an overall count balance among the obtained sncRNA data; thus, a decrease in one sncRNA abundance would lead to an increase in other sncRNAs, even if their absolute copy numbers remain unchanged. Given this interdependence introduced by relative quantification, we therefore analyzed the co-expression patterns among different sncRNA types across the NZS, AZS, and TZS groups, which might indicate the regulatory interactions or compositional homeostasis of different sperm sncRNAs. The data showed that different subtypes of sncRNAs exhibit unique expression correlation signatures across the NZS, AZS, and TZS groups. Interestingly, the magnitude of differential expression did not always correspond to the extent of disruption observed in its co-expression network. For example, although miRNAs showed pronounced expression alterations in the AZS and TZS groups, their intra-group co-expression patterns remained similar to those observed in normal sperm (Fig. 4C–H). This discrepancy suggests that sncRNA co-expression networks may possess a certain level of robustness, and although individual expression levels fluctuate, their co-expression regulatory relationships may remain relatively stable. Nevertheless, such co-expression pattern analyses offer a systematic perspective on how different sncRNAs might interact or be stabilized within the human sperm milieu, beyond individual expression changes.

While miRNA-based diagnostics have been explored in other disease contexts,^42^ the diagnostic potential of rsRNAs and tsRNAs, especially in the context of human fertility assessment, remains largely uncharted. In this study, we explored the diagnostic potential of tsRNAs and rsRNAs in human sperm quality evaluation based on their expression signatures across the NZS, AZS, and TZS groups. We developed multiple diagnostic classifiers (MSsncSig, AZSsncSig, and TZSsncSig) based on sperm rsRNA and tsRNA profiles. These classifiers, consisting of panels with selective sperm small non-coding RNAs, demonstrated superior diagnostic performance compared with conventional clinical semen parameters, exhibiting significant correlations with critical fertility parameters and strong diagnostic accuracy in differentiating subfertile individuals from healthy controls. However, the clinical implications of these findings should be interpreted cautiously given the limited sample size (*n* = 25) in this study. Validation in larger, independent cohorts by PANDORA-seq, or quantitative reverse transcription PCR and microfluidic-based assays adapted for rapid and cost-effective detection of tsRNA and rsRNAs in semen samples, is necessary to further optimize these biomarkers and support their translational application into standardized clinical diagnostic tools. Nevertheless, the consistent presence of sperm rsRNA and tsRNA expression patterns across normal and subfertility groups suggests their clinical application potential as diagnostic biomarkers and provides new insights for researchers to investigate their functional contributions in spermatogenesis, fertilization, and embryonic development, which would further expand their potential utility as a toolbox of clinical male fertility evaluation.

## CRediT authorship contribution statement

**Ruofan Huang:** Writing – original draft, Methodology, Formal analysis, Conceptualization. **Yiting Yang:** Writing – original draft, Validation, Methodology, Conceptualization. **Wenlin Jiang:** Writing – review & editing. **Zheng Cao:** Writing – review & editing. **Junchao Shi:** Writing – review & editing, Formal analysis. **Xiao-Ou Zhang:** Writing – original draft, Formal analysis, Conceptualization. **Yunfang Zhang:** Writing – original draft, Methodology, Conceptualization.

## Data availability

The data underlying this article are available in the article and in its online supplementary data, and in the Genome Sequence Archive for Human at https://www.cncb.ac.cn, and can be accessed with PRJCA031725.

## Funding

This work was supported by the 10.13039/501100012166National Key Research and Development Program of China (No. 2019YFA0802600 to Y.F.Z.; 2022YFA1103200 to X.O.Z.), the 10.13039/501100001809National Natural Science Foundation of China (No. 82371727, 82022029, 81971460 to Y.F.Z.; 32170553 to X.O.Z.; 32370596 to J.C.S.), 10.13039/501100003399Science and Technology Commission of Shanghai Municipality, China (No. 23JC1403802 to Y.F.Z.), Innovation Promotion Program of NHC and Shanghai Key Labs, SIBPT (China) (No. RC2024-04 to Y.T.Y.), Strategic Priority Research Program of the Chinese Academy of Sciences (No. XDA0460302 to J.C.S.), and 10.13039/501100012226Fundamental Research Funds for the Central Universities of China (No. 22120240435 to Y.F.Z. and X.O.Z.). The 10.13039/501100012166National Key Research and Development Program of China provides the funding for the open access charge.

## Conflict of interests

Yunfang Zhang is an Editorial Board member for *Genes & Diseases* and was not involved in the editorial review or the decision to publish this article. All authors declare that there are no competing interests.

## References

[1] Rotella A., Varnum M.E.W., Sng O., Grossmann I. (2021). Increasing population densities predict decreasing fertility rates over time: a 174-nation investigation. Am Psychol.

[2] Aitken R.J. (2022). The changing tide of human fertility. Hum Reprod.

[3] Houston B.J., Riera-Escamilla A., Wyrwoll M.J. (2021). A systematic review of the validated monogenic causes of human male infertility: 2020 update and a discussion of emerging gene-disease relationships. Hum Reprod Update.

[4] Niederberger C. (2021). Male infertility. J Urol.

[5] Lu J.C., Huang Y.F., Lü N.-Q. (2010). WHO laboratory manual for the examination and processing of human semen: its applicability to andrology laboratories in China. Zhonghua Nan ke Xue.

[6] Oehninger S., Ombelet W. (2019). Limits of current male fertility testing. Fertil Steril.

[7] Smorag L., Zheng Y., Nolte J., Zechner U., Engel W., Pantakani D.V. (2012). microRNA signature in various cell types of mouse spermatogenesis: evidence for stage-specifically expressed miRNA-221,-203 and-34b-5p mediated spermatogenesis regulation. Biol Cell.

[8] Gan M., Jing Y., Xie Z. (2023). Potential function of testicular microRNAs in heat-stress-induced spermatogenesis disorders. Int J Mol Sci.

[9] Girard A., Sachidanandam R., Hannon G.J., Carmell M.A. (2006). A germline-specific class of small RNAs binds mammalian Piwi proteins. Nature.

[10] Gou L.T., Dai P., Yang J.H. (2014). Pachytene piRNAs instruct massive mRNA elimination during late spermiogenesis. Cell Res.

[11] Pantano L., Jodar M., Bak M. (2015). The small RNA content of human sperm reveals pseudogene-derived piRNAs complementary to protein-coding genes. RNA.

[12] Peng H., Shi J., Zhang Y. (2012). A novel class of tRNA-derived small RNAs extremely enriched in mature mouse sperm. Cell Res.

[13] Yu Y., Gu J., Jin Y. (2015). Panoramix enforces piRNA-dependent cotranscriptional silencing. Science.

[14] Zhang Y., Zhang X., Shi J. (2018). Dnmt2 mediates intergenerational transmission of paternally acquired metabolic disorders through sperm small non-coding RNAs. Nat Cell Biol.

[15] Zhao S., Gou L.T., Zhang M. (2013). piRNA-triggered MIWI ubiquitination and removal by APC/C in late spermatogenesis. Dev Cell.

[16] Zimmermann C., Romero Y., Warnefors M. (2014). Germ cell-specific targeting of DICER or DGCR8 reveals a novel role for endo-siRNAs in the progression of mammalian spermatogenesis and male fertility. PLoS One.

[17] Chen Q., Yan M., Cao Z. (2016). Sperm tsRNAs contribute to intergenerational inheritance of an acquired metabolic disorder. Science.

[18] Chen X., Zheng Y., Lei A. (2020). Early cleavage of preimplantation embryos is regulated by tRNA(Gln-TTG)-derived small RNAs present in mature spermatozoa. J Biol Chem.

[19] Aravin A., Gaidatzis D., Pfeffer S. (2006). A novel class of small RNAs bind to MILI protein in mouse testes. Nature.

[20] Sharma U., Conine C.C., Shea J.M. (2016). Biogenesis and function of tRNA fragments during sperm maturation and fertilization in mammals. Science.

[21] Gou L.T., Kang J.Y., Dai P. (2017). Ubiquitination-deficient mutations in human piwi cause male infertility by impairing histone-to-protamine exchange during spermiogenesis. Cell.

[22] Liu B., Cao J., Wang X., Guo C., Liu Y., Wang T. (2021). Deciphering the tRNA-derived small RNAs: origin, development, and future. Cell Death Dis.

[23] Chen Q., Zhang X., Shi J., Yan M., Zhou T. (2021). Origins and evolving functionalities of tRNA-derived small RNAs. Trends Biochem Sci.

[24] Li Y., Yu Z., Jiang W. (2024). tRNA and tsRNA: from heterogeneity to multifaceted regulators. Biomolecules.

[25] Lambert M., Benmoussa A., Provost P. (2019). Small non-coding RNAs derived from eukaryotic ribosomal RNA. Noncoding RNA.

[26] Shi J., Zhang Y., Tan D. (2021). *PANDORA*-seq expands the repertoire of regulatory small RNAs by overcoming RNA modifications. Nat Cell Biol.

[27] Chen L., Xu W., Liu K. (2021). 5' half of specific tRNAs feeds back to promote corresponding tRNA gene transcription in vertebrate embryos. Sci Adv.

[28] Di Fazio A., Schlackow M., Pong S.K., Alagia A., Gullerova M. (2022). Dicer dependent tRNA derived small RNAs promote nascent RNA silencing. Nucleic Acids Res.

[29] Choi E.J., Ren J., Zhang K. (2020). The importance of AGO 1 and 4 in post-transcriptional gene regulatory function of tRF5-GluCTC, an respiratory syncytial virus-induced tRNA-derived RNA fragment. Int J Mol Sci.

[30] Guzzi N., Cieśla M., Ngoc P.C.T. (2018). Pseudouridylation of tRNA-derived fragments steers translational control in stem cells. Cell.

[31] Shi J., Zhang Y., Zhou T., Chen Q. (2019). tsRNAs: the Swiss army knife for translational regulation. Trends Biochem Sci.

[32] Kim H.K., Fuchs G., Wang S. (2017). A transfer-RNA-derived small RNA regulates ribosome biogenesis. Nature.

[33] Zhang Y., Shi J., Chen Q. (2017). tsRNAs: new players in mammalian retrotransposon control. Cell Res.

[34] Yu J., Zhang X., Cai C., Zhou T., Chen Q. (2025). Small RNA and toll-like receptor interactions: origins and disease mechanisms. Trends Biochem Sci.

[35] Ko E.A., Zhou T., Ko J.H. (2024). Insight into noncanonical small noncoding RNAs in Influenza A virus infection. Virus Res.

[36] Cozen A.E., Quartley E., Holmes A.D., Hrabeta-Robinson E., Phizicky E.M., Lowe T.M. (2015). ARM-seq: alkb-facilitated RNA methylation sequencing reveals a complex landscape of modified tRNA fragments. Nat Methods.

[37] Dai Q., Zheng G., Schwartz M.H., Clark W.C., Pan T. (2017). Selective enzymatic demethylation of N_2_, N(2)-dimethylguanosine in RNA and its application in high-throughput tRNA sequencing. Angew Chem Int Ed.

[38] Zhang X., Trebak F., Souza L.A.C. (2020). Small RNA modifications in Alzheimer’s disease. Neurobiol Dis.

[39] Chen Q., Yan W., Duan E. (2016). Epigenetic inheritance of acquired traits through sperm RNAs and sperm RNA modifications. Nat Rev Genet.

[40] Shi J., Ko E.A., Sanders K.M., Chen Q., Zhou T. (2018). SPORTS1.0: a tool for annotating and profiling non-coding RNAs optimized for rRNA- and tRNA-derived small RNAs. Genomics Proteomics Bioinformatics.

[41] Zhang Y., Shi J., Rassoulzadegan M., Tuorto F., Chen Q. (2019). Sperm RNA code programmes the metabolic health of offspring. Nat Rev Endocrinol.

[42] Metcalf G.A.D. (2024). microRNAs: circulating biomarkers for the early detection of imperceptible cancers via biosensor and machine-learning advances. Oncogene.

