# Supplementary material for: PANDORA-seq reveals human sperm sncRNA signature endowed with sperm quality assessment
Source: Genes Dis. 2025 Aug 18;13(4):101807. doi: 10.1016/j.gendis.2025.101807 (PMC13092018; doi:10.1016/j.gendis.2025.101807)
Supplement: Multimedia component 1 [file mmc1.docx]

**Supplementary Material**


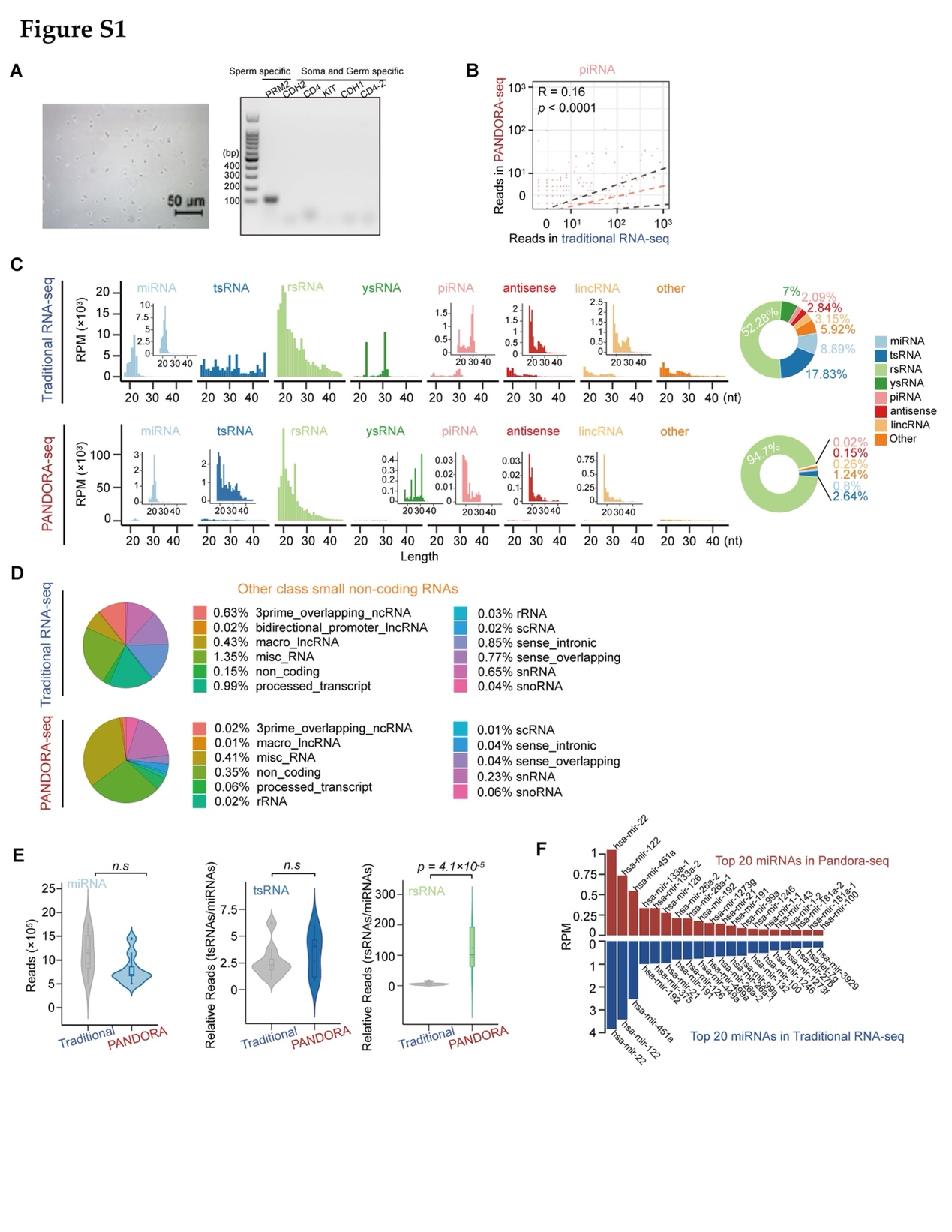


**Figure S1. Human sperm sncRNA profiles revealed by PANDORA-seq and traditional sncRNA-seq.** (A) Light microscope image of purified human sperm. (Left) The purity of human sperm was evaluated by RT-PCR using specific molecular markers. *PRM2*, protamine 2; *CD4*, CD4 molecule; *CDH1*, cadherin 1; *CD4-2*, CD4 molecule; C*DH2*, cadherin 2; *KIT*, KIT proto-oncogene, receptor tyrosine kinase, also known as *C-KIT.* (Right) (B) Scatter plots depicting the correlation of piRNA expression profiles of paired traditional sncRNA-seq and PANDORA-seq. Spearman correlation coefficients and p-values are shown. (C) Dynamic landscapes and length distributions of eight major sncRNA categories detected by traditional sncRNA-seq and PANDORA-seq protocols (n = 9). Zoomed panels of sncRNAs are shown on the plot. (D) Relative expression proportion of sncRNAs belonging to the ‘other’ group in healthy human sperm. The ‘other’ group includes sncRNAs derived from snoRNAs, snRNAs, processed transcripts, and other noncoding transcripts. (E) Violin plots comparing the relative expression levels of total miRNAs, tsRNAs/miRNAs, rsRNAs/miRNAs by traditional sncRNA-Seq and PANDORA-seq. Wilcoxon rank-sum test p-values are shown. (F) The top 20 miRNAs detected by traditional sncRNA-seq (blue bars) against PANDORA-seq (red bars), ordered by RPM.


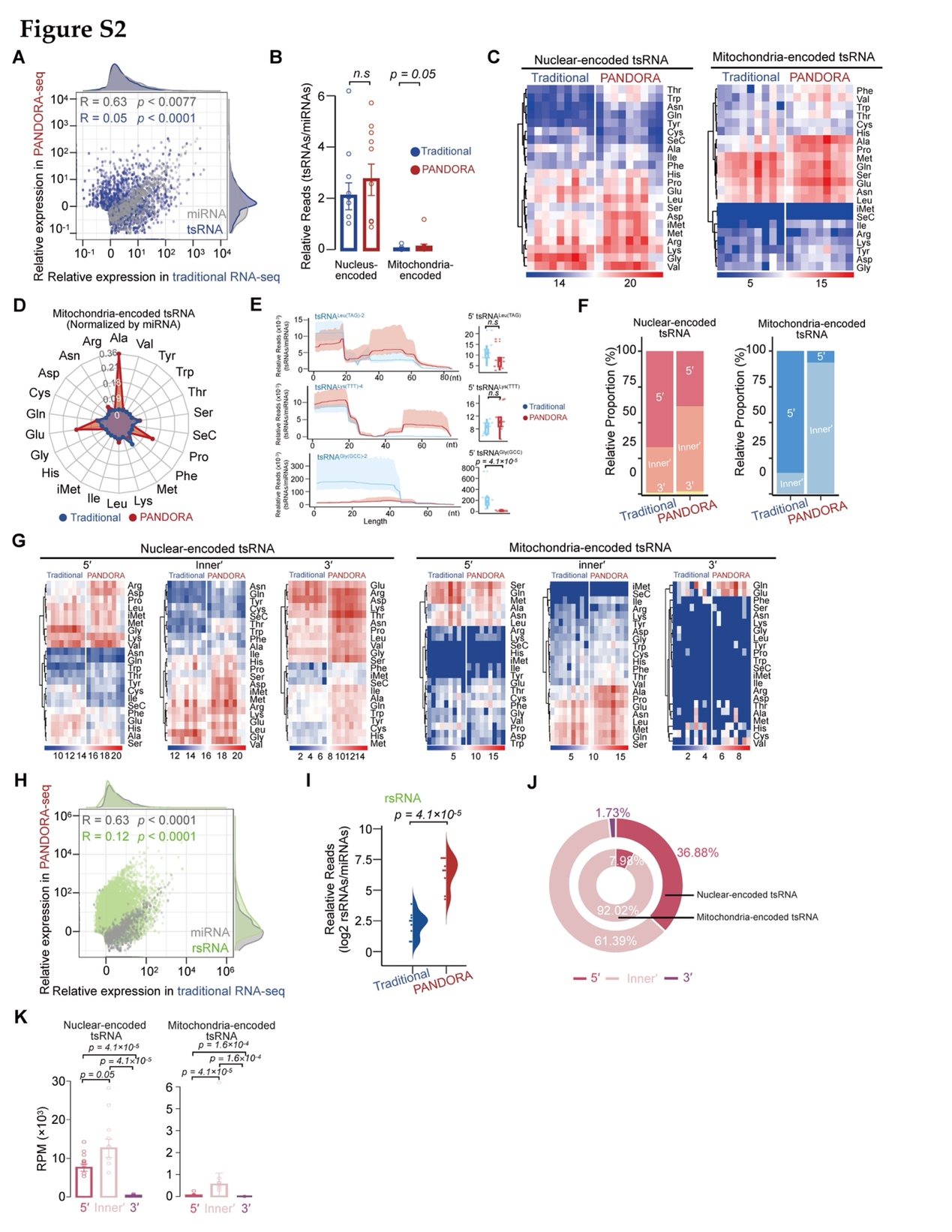


**Figure S2. Different types of sncRNA abundances in human sperm revealed by PANDORA-Seq and traditional sncRNA-seq.** (A) Scatter plots comparing profile changes in tsRNAs (blue) and miRNAs (grey) detected using traditional sncRNA-seq versus PANDORA-seq protocols. Spearman correlation coefficients and p-values are shown. (B) Bar charts showing the relative expression levels of tsRNAs (genomic and mitochondrial), normalized to total miRNA levels, detected by traditional sncRNA-seq and PANDORA-seq. (C) Heatmaps showing the nucleus-encoded and mitochondrial tsRNA relative expression levels (normalized to total miRNA levels and based on a log2-transformed scale in the row direction) in healthy human sperm, as detected by traditional sncRNA-seq and PANDORA-seq. (D) Radar plot showing the different relative expression proportions of each mitochondria-encoded tsRNA subcategory with respect to these two protocols. (E) Sequence mapping location and expression profile of tsRNA^Leu(TAG)^, tsRNA^Lys(TTT)^, and tsRNA^Gly(GCC)^ in human sperm. (F) Proportional distribution of the three distinct origins (5', inner', and 3') of nucleus-encoded and mitochondrial-encoded tsRNAs. (G) Heatmaps showing the relative expression levels of nucleus-encoded and mitochondria-encoded tsRNA origins (normalized to total miRNA levels and based on a log2-transformed scale in the row direction) for each tsRNA subcategory. (H) Scatter plot comparing profile changes in rsRNAs (green) and miRNAs (grey) detected by traditional sncRNA-seq and PANDORA-seq. Spearman correlation coefficients and p-values are shown. (I) Raincloud plot comparing the relative expression levels of rsRNAs (normalized to total miRNA levels and based on a log2-transformed scale) in healthy human sperm by traditional sncRNA-seq and PANDORA-seq. (J) Donut chart showing the relative abundances of four distinct tsRNA origins (5', inner', and 3') for cyto-tsRNAs and mt-tsRNAs. (K) Bar charts depicting the relative abundances of three distinct tsRNA origins (5', inner', and 3') for both cyto-tsRNAs and mt-tsRNAs. Wilcoxon rank-sum test p-values are shown.


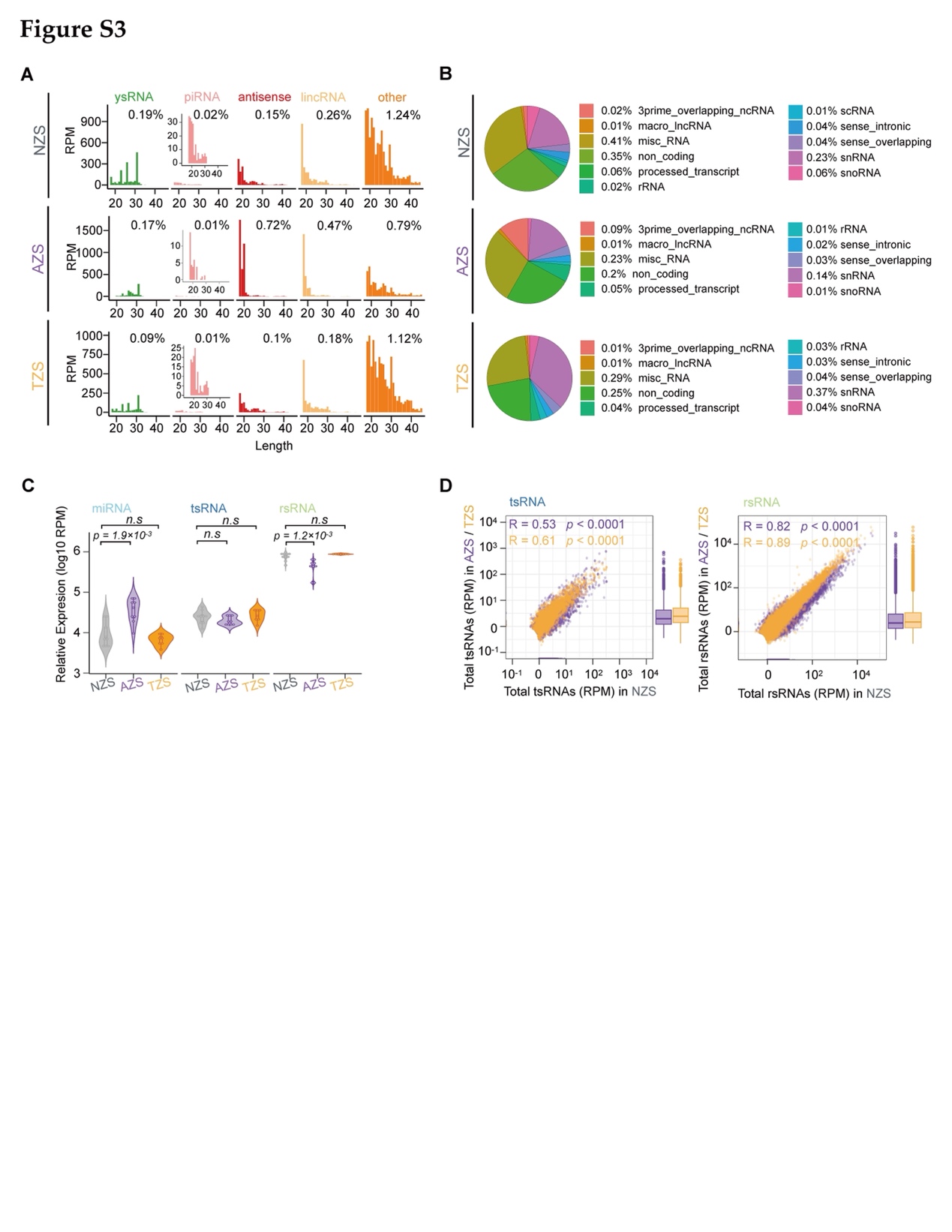


**Figure S3. SncRNAs alteration signatures in NZS, AZS, and TZS.** (A) Length distributions of other major sncRNA categories among NZS, AZS, and TZS. Zoomed panels of piRNAs are shown on the plot. (B) Relative expression proportion of sncRNAs belonging to the ‘other’ group among NZS, AZS, and TZS samples. The ‘other’ group includes sncRNAs derived from snoRNAs, snRNAs, processed transcripts, and other noncoding transcripts. (C) Comparison of the relative expression levels of three major sncRNA types (miRNA, tsRNA, rsRNA, and ysRNA) across NZS, AZS, and TZS groups. (D) Scatter plots depicting the correlation of tsRNA and rsRNA expression profiles between subfertile sperm (AZS or TZS) and healthy control (NZS) samples. Spearman correlation coefficients and p-values are shown.


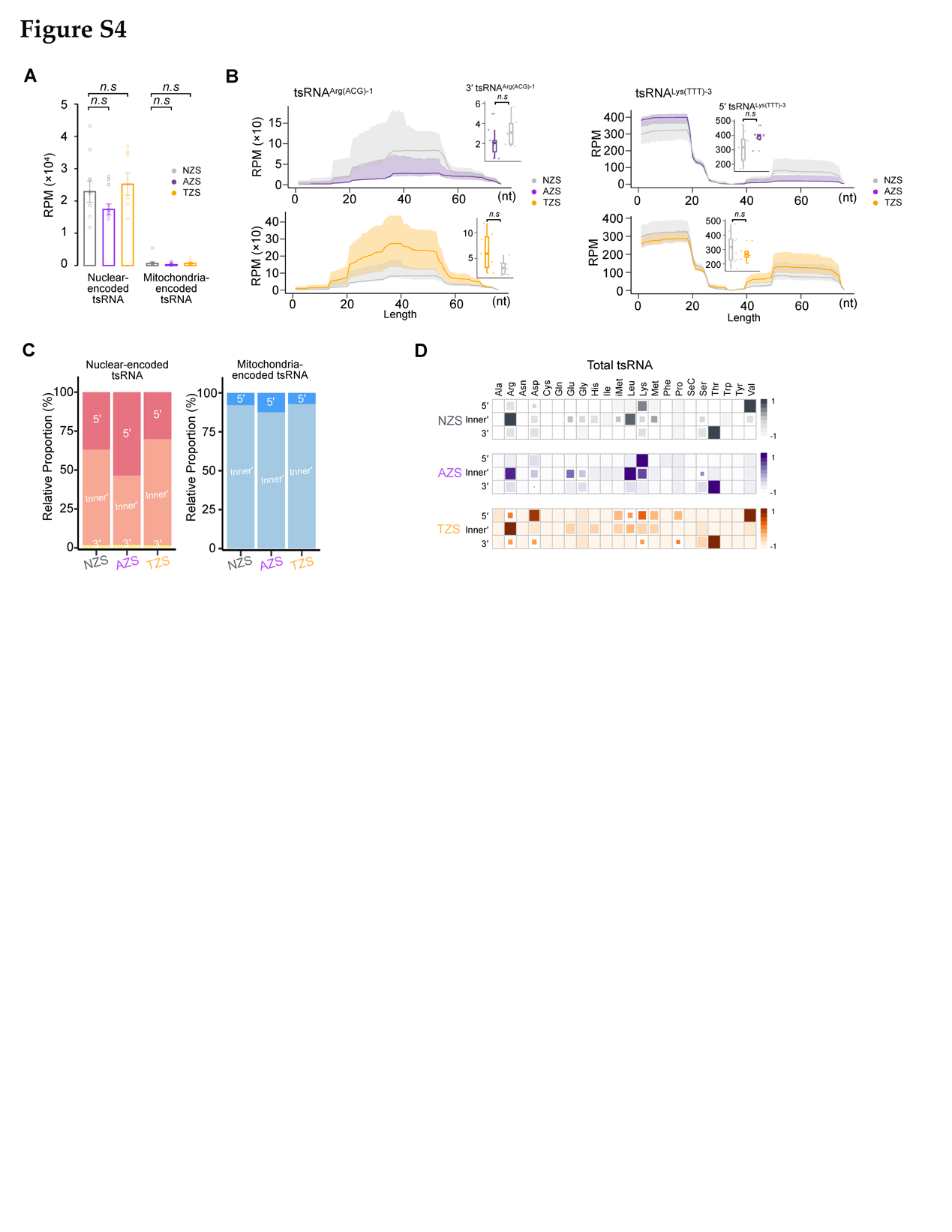


**Figure S4. The tsRNAs signatures in NZS, AZS, and TZS.** (A) Bar charts depicting the proportional distribution of cyto-tsRNAs and mt-tsRNAs among NZS, AZS, and TZS samples. (B) Sequence mapping location and expression profile of tsRNA^Arg (ACG)^ and tsRNA^Lys(TTT)^ in samples. (C) Proportional distribution of the three distinct origins (5', inner', and 3') of nucleus-encoded and mitochondrial-encoded tsRNAs among NZS, AZS, and TZS samples. (D) Heatmap representing the expression abundances of each tsRNA subcategory on a min-max scaled transformation across three distinct tsRNA origins among NZS, AZS, and TZS samples.


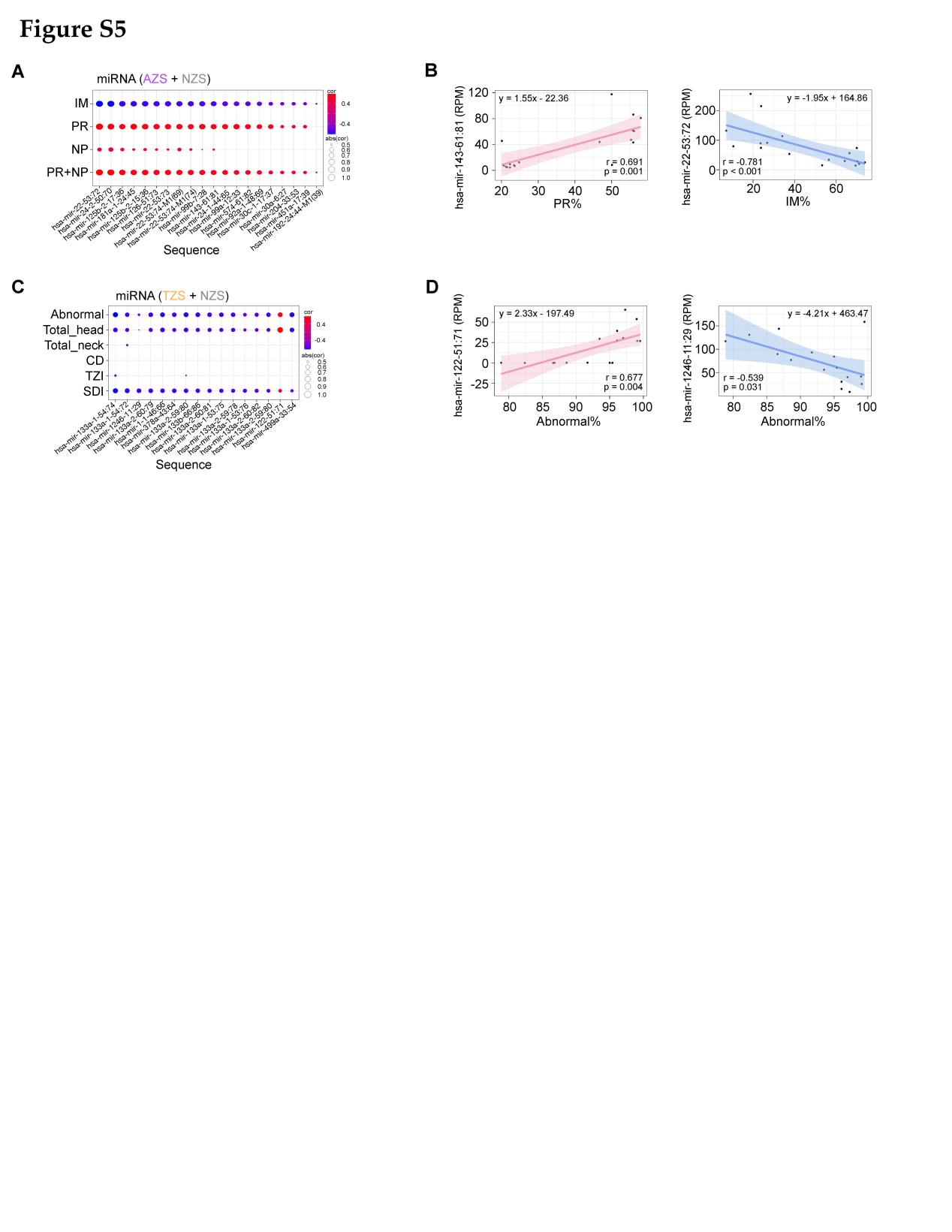


**Figure S5. The miRNAs signatures in NZS, AZS, and TZS.** (A-D) Dot Plot of sperm motility (A) or sperm morphology metrics (C) correlated with individual miRNA expression levels. Linear correlations between representative miRNAs and sperm motility (B) or sperm morphology metrics (D).


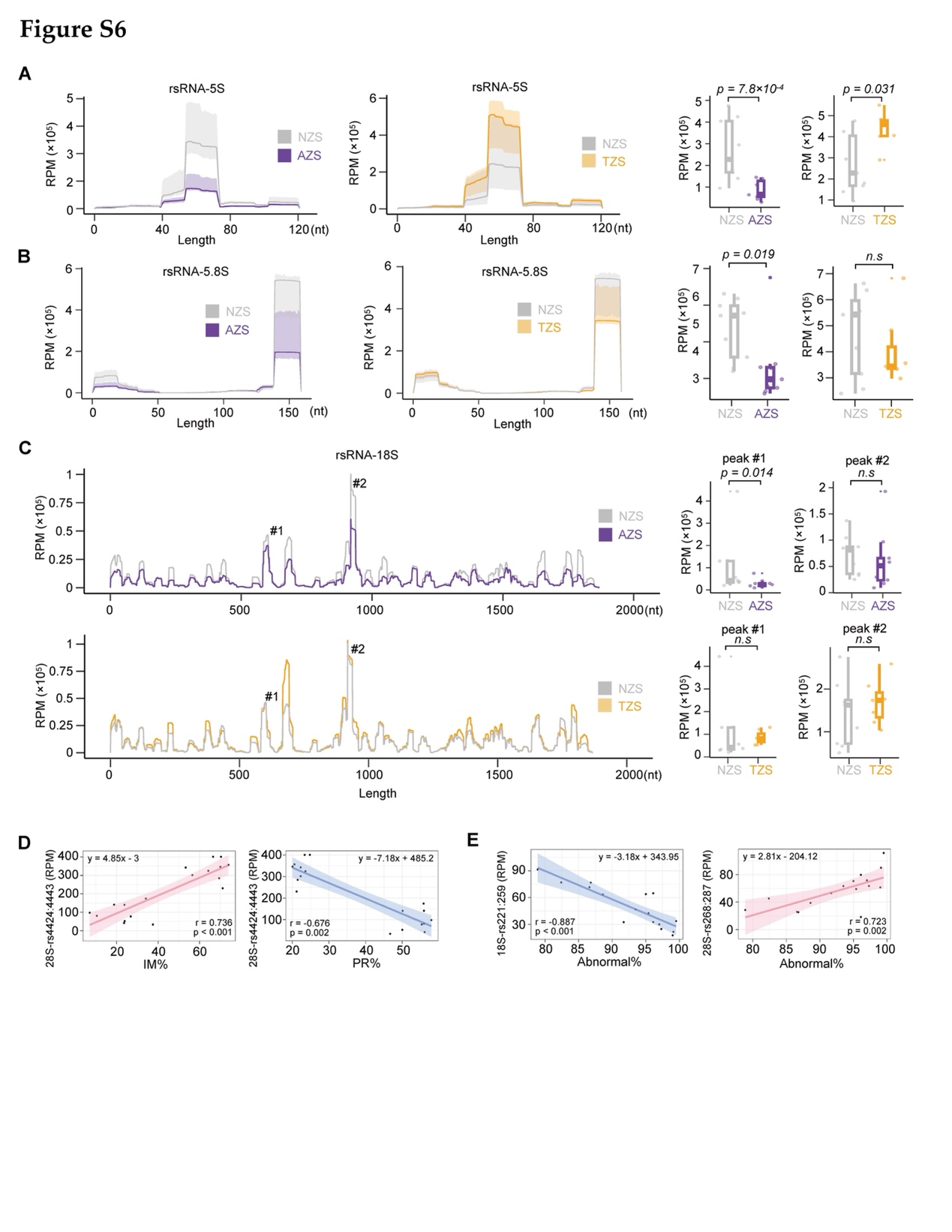


**Figure S6. The rsRNAs signatures in NZS, AZS, and TZS.** (A-C) Sequence mapping location and expression profile of rsRNA-5S (A), rsRNA-5.8S (B), rsRNA-18S (C) between subfertile sperm (AZS or TZS) and healthy control (NZS) samples. (D-E) Linear correlations between representative rsRNA and sperm motility (D) or sperm morphology metrics (E).


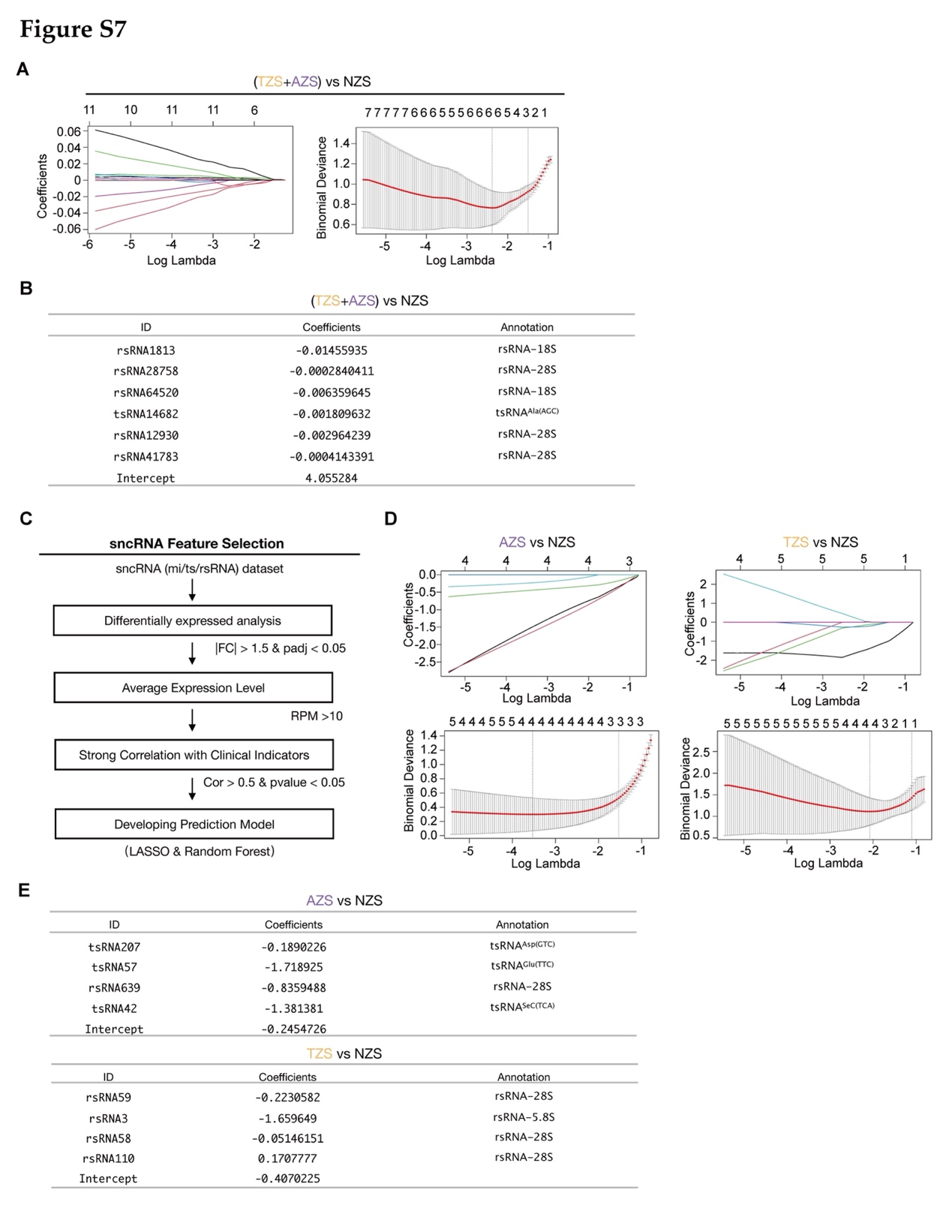


**Figure S7. Identification of the specific sncRNA signatures in NZS, AZS, and TZS by LASSO screening.** (A) Schematic diagram of LASSO screening and the binomial deviance diagram for the classification model between subfertile sperm (AZS and TZS) and healthy control (NZS) samples. (B) A list of sncRNAs that composed the classification model with annotations between subfertile sperm (AZS and TZS) and healthy control (NZS) samples. (C) Strategies and workflow of filtering sncRNA characteristics for the classification model. (D) Schematic diagram of LASSO screening in two different comparing groups. (E) A list of sncRNAs that composed the classification model with annotations.

**Table S1:**

Primers：

| hPRM2-F | sperm-specific gene | GGATCCACAGGCGGCAGCATCGCT |
| --- | --- | --- |
| hPRM2-R |  | GCATGTTCTCTTCCTGGTTCTGCA |
| hCDH1-F | epithelial cell markers | CACCTTCCATGACAGACCC |
| hCDH1-R |  | AACGCATTGCCACATACAC |
| hCDH2-F |  | CTGAAGTGACTCGTAACGACG |
| hCDH2-R |  | CATGTCAGCCAGCTTCTTGAAG |
| hCD4-F1 | a leukocyte T-cell surface marker | TTCAACTGTAAAGGCGAGTG |
| hCD4-R1 |  | CGGATTGACTGCCAACTCT |
| hCD4-F2 |  | GTGAACCTGGTGGTGATGAGAGC |
| hCD4-R2 |  | GGCTACATGTCTTCTGAAACCGGTG |
| hC-KIT-F | germline-specific gene | TACAACGATGTGGGCAAGA |
| hC-KIT-R |  | TACGAAACCAATCAGCAAAG |

Rt-PCR Procedure：

| 94℃ | 5min |  |
| --- | --- | --- |
| 94℃ | 30s | *35 |
| 58/60℃ | 30s |  |
| 72℃ | 30s |  |
| 72℃ | 5min |  |

**Table S2**

T4PNK solution:

| 10x PNK Buffer | 2 μl |
| --- | --- |
| T4 PNK | 1 μl |
| 10mM ATP | 1 μl |
| RNA | 10 μl |
| NF H_2_O | 6 μl |
| Total | 20 μl |

AlkB solution:

| 20x HEPES | 5 μl |
| --- | --- |
| 20x Fe2+ | 5 μl |
| 10x aK | 10 μl |
| 10x SA | 10 μl |
| AlkB | 2 μl |
| RRI | 4 μl |
| NF H2O | 44 μl |
| Total | 100ul |

**Table S3:**

Probe sequences:

| mir-122 | AAACACCATTGTCACACTCCA | 5′-DIG |
| --- | --- | --- |
| tsRNA^Ala(AGC)^ | TGGAGAATGTGGGCATCGATCCCACT | 5′-DIG |
| tsRNA^Leu(TAG)^ | CCTTAGACCGCTCGGCCACACTACC | 5′-DIG |
| tsRNA^Lys(TTT)^ | GCTCTACCGACTGAGCTATCCGGGC | 5′-DIG |
| tsRNA^Gly(GCC)^ | TCTACCACTGAACCACCAAT | 5′-DIG |
| tsRNA^Arg(ACG)^ | TCGACTCCTGGCTGGCTCGCCA | 3′-DIG |
| rsRNA-5.8S | AGCGACGCTCAGACAGGCGT | 5′-DIG |
| rsRNA-18S #1 | CCGCTCCCAAGATCCAACTACGAGC | 5′-DIG |
| rsRNA-18S #2 | GCCCCCGGCCGTCCCTCTTAATCA | 5′-DIG |
| rsRNA-28S #1 | ACCACCCGCTTTGGGCTGCATTCCC | 5′-DIG |
| rsRNA-28S #2 | TCGCGCACGTGTTAGACTCCTTGG | 5′-DIG |

Primer:

| miRNA-34C | AGGCAGTGTAGTTAGCTGATTGC |
| --- | --- |
| rsRNA-18S #1 | AAAAAGCTCGTAGTTGGATCT |
| rsRNA-18S #2 | TGATTAAGAGGGACGGCCGGG |
| rsRNA-28S #1 | GGGAATGCAGCCCAAAGCGGG |
| rsRNA-28S #2 | CCAAGGAGTCTAACACGTGCG |
| 5′ tsRNA^Lys(TTT)^ | GCCCGGCTAGCTCAGTCGGTAGAGCATG |
| Inner′ tsRNA^Lys(TTT)^ | AGACTTTTAATCTGAGGGTC |
| 3′ tsRNA^Lys(TTT)^ | TTCAAGTCCCTGTTCGGGCG |
| 5′ tsRNA^Ala(AGC)^ | GGGGGTGTAGCTCAGTGGTAGAGCG |
| Inner′ tsRNA^Ala(AGC)^ | GAGGTAGTGGGATCGATGCC |
| 3′ tsRNA^Ala(AGC)^ | ATCGATGCCCACATTCTCCA |
| 5′ tsRNA^Glu(CTC)^ | TCCCTGGTGGTCTAGTGGTT |
| Inner′ tsRNA^Glu(CTC)^ | ACCGCCGCGGCCCGGGTT |
| 3′ tsRNA^Glu(CTC)^ | CGATTCCCGGTCAGGGAA |
| 5′ tsRNA^Arg(CCT)^ | CCTAAGCCAGGGATTGTGGGTTCGAGT |
| Inner′ tsRNA^Arg(CCT)^ | AAGGCGTCTGACTTCGGATC |
| 3′ tsRNA^Arg(CCT)^ | TCGAGTCCTGCCGCGGTCG |
| 5′ tsRNA^Val(AAC)^ | GTTTCCGTAGTGTAGTGGTT |
| Inner′ tsRNA^Val(AAC)^ | GTTCGCCTAACACGCGAAAG |
| 3′ tsRNA^Val(AAC)^ | TTCGAAACCGGGCGGAAACA |
